# The Time Trend Temperature–Mortality as a Factor of Uncertainty Analysis of Impacts of Future Heat Waves: Wu et al. Respond

**DOI:** 10.1289/ehp.1308042R

**Published:** 2014-05-01

**Authors:** Jianyong Wu, Ying Zhou, Yang Gao, Joshua S. Fu, Brent A. Johnson, Cheng Huang, Young-Min Kim, Yang Liu

**Affiliations:** 1 Department of Environmental Health, Rollins School of Public Health, Emory University, Atlanta, Georgia, USA; 2 Department of Civil and Environmental Engineering, University of Tennessee, Knoxville, Tennessee, USA; 3 Atmospheric Science and Global Change Division, Pacific Northwest National Laboratory, Richland, Washington, USA; 4 Department of Biostatistics and Bioinformatics, Rollins School of Public Health, Emory University, Atlanta, Georgia, USA; 5 Department of Global Health, School of Public Health and Health Services, George Washington University, Washington, DC, USA

We thank Linares et al. for their interest in our article and for broadening the discussion on the uncertainties in predicting the health impact of future heat waves. Linares et al. pointed out that the possible evolution over time can take place both in minimum mortality temperatures related to heat waves and in the modifications of these possible impacts due to socioeconomic improvements. Although such considerations were beyond the scope of our published analysis (Wu et al. 2014), we agree that socioeconomic and demographic factors can have profound impacts on the estimated excess mortality in a changing climate.

A heat wave is defined as a period of consecutive days with temperatures exceeding a certain threshold based on physiologic effects (Robinson 2001). The threshold temperature is usually calculated based on local historical data, which can vary in both time and space. Linares et al. suggested that heat wave definition temperatures might be reduced to a consequence of population aging in time. Given these changes in the threshold temperature over time, the heat wave definition would indeed add an additional layer of uncertainty to the predicted health impact of future heat waves on top of what we have characterized in the paper. Such uncertainty, however, is difficult to quantify without detailed data on the structure of future populations, especially age. So far, the U.S. Census Bureau (2012) has issued only national-level, age-specific population projections.

The health impacts of heat waves can be modified by many factors, such as race, age, sex, socioeconomic status, and geographic location (Hajat and Kosatky 2010). The changing impacts of heat waves on cardiovascular/circulatory and respiratory mortality (Ha and Kim, 2013; Mirón et al. 2008) seem to be related to the improvements in health care services and living conditions over time. These trends may be generalizable in space if we are willing to assume that the U.S. health care system has improved its service to cardiovascular patients over the years in a fashion similar to that of Spain, Italy, or other developed countries. However, it may not be justifiable to extrapolate them in time because the impact of these improvements is likely to taper off unless significant technological advancement takes place in the future.

In addition, early warning systems and adaptation strategies can strongly influence the impact of heat waves on a society (Lowe et al. 2011). However, the relative risk of heat waves must be estimated using existing health data records, making it very difficult to take any adaptation measures into consideration because we lack such examples in the past. In our study, we set future baseline mortality rate and relative health risk of heat waves as constant because robust estimates of these parameters for the 2050s are unavailable. Further research is needed to address these issues in order to provide a more comprehensive and realistic evaluation of the impact of future heat waves.
